# Trajectories of work disability and unemployment among young adults with common mental disorders

**DOI:** 10.1186/s12889-018-6141-y

**Published:** 2018-11-06

**Authors:** Magnus Helgesson, Petter Tinghög, Mo Wang, Syed Rahman, Fredrik Saboonchi, Ellenor Mittendorfer-Rutz

**Affiliations:** 1 Division of Insurance Medicine, Department of Clinical Neuroscience, Karolinska Institutet, SE-171 77 Stockholm, Sweden; 2 The Swedish Red Cross University, College, SE-102 15 Stockholm, Sweden

**Keywords:** Sick leave, Disability pension, Unemployment, Common mental disorders, Labour market marginalisation, Education

## Abstract

**Background:**

Labour-market marginalisation (LMM) and common mental disorders (CMDs) are serious societal problems. The aims were to describe trajectories of LMM (both work disability and unemployment) among young adults with and without CMDs, and to elucidate the characteristics associated with these trajectories.

**Methods:**

The study was based on Swedish registers and consisted of all individuals 19–30 years with an incident diagnosis of a CMD in year 2007 (*n* = 7245), and a matched comparison group of individuals without mental disorders during the years 2004–07 (*n* = 7245). Group-based trajectory models were used to describe patterns of LMM both before, and after the incident diagnosis of a CMD. Multinomial logistic regressions investigated the associations between sociodemographic and medical covariates and the identified trajectories.

**Results:**

Twenty-six percent (*n* = 1859) of young adults with CMDs followed trajectories of increasing or constant high levels of work disability, and 32 % (*n* = 2302) followed trajectories of increasing or constant high unemployment. In the comparison group, just 9 % (*n* = 665) followed increasing or constant high levels of work disability and 21 % (*n* = 1528) followed trajectories of increasing or constant high levels of unemployment. A lower share of young adults with CMDs followed trajectories of constant low levels of work disability (*n* = 4546, 63%) or unemployment (*n* = 2745, 38%), compared to the level of constant low work disability (*n* = 6158, 85%) and unemployment (*n* = 3385, 50%) in the comparison group. Remaining trajectories were fluctuating or decreasing. Around 50% of young adults with CMDs had persistent levels of LMM at the end of follow-up. The multinomial logistic regression revealed that educational level and comorbid mental disorders discriminated trajectories of work disability, while educational level, living area and age determined differences in trajectories of unemployment (R^2^_difference_ = 0.02–0.05, *p* < 0.001).

**Conclusions:**

A large share, nearly 50%, of young adults with CMDs, substantially higher than in the comparison group of individuals without mental disorders, display increasing or high persistent levels of either work disability or unemployment throughout the follow-up period. Low educational level, comorbidity with other mental disorders and living in rural areas were factors that increased the probability for LMM.

**Electronic supplementary material:**

The online version of this article (10.1186/s12889-018-6141-y) contains supplementary material, which is available to authorized users.

## Background

Labour market marginalisation (LMM) is a serious societal problem among young adults with common mental disorders (CMDs), i.e. depressive, anxiety and stress-related disorders, in many Western countries [1]. Due to the early onset and the risk of frequent relapses, as many as 20% of the population in working age is at any given time estimated to fulfil the criteria for being diagnosed with a mental disorder, the vast majority with CMDs [1]. Young adults with CMDs are at particular risk of work disability [1–4], and/or unemployment [3, 5, 6], and may face considerable challenges to independently support themselves through gainful employment. This may imply huge challenges for societies, as the costs for loss of production and welfare benefits will increase significantly. Periods of work disability and unemployment might also in itself further deteriorate health [7–9]. To date, different definitions of LMM exist, and previous studies have shown that there is a risk to underestimate the true consequences of CMDs if LMM is defined only as unemployment [8, 10]. This study has therefore conceptualised LMM from a social insurance perspective and included measures both based on medical assessments (work disability in terms of sickness absence and disability pension) and measures not based on medical assessments (unemployment).

There are to date several studies with regard to mental disorders, and subsequent LMM, but very few that have a sole focus on young adults, a group with most of their working life ahead of them [11, 12]. There is particularly a lack of studies that can elucidate the presumed heterogeneity of patterns of LMM both *before* and *after* an incident diagnosis of a CMD. In order to shed light on the potential downward spiral among young adults with CMDs, studies that can elucidate the complex relation between CMDs and LMM longitudinally are warranted.

In order to have a basis for the design of future intervention studies, it is crucial to investigate how heterogeneous patterns of LMM are characterised by different sociodemographic and medical factors. Here, several sociodemographic factors are reported to be associated with an increased risk of LMM, as low educational level, migration background and residence outside big cities [13–17]. Moreover, comorbidity with other mental and somatic disorders, e.g. substance abuse and personality disorders may further decrease the work capacity and are common among young adults with CMDs [18, 19]. We have in this study used group based trajectory models, which have the inherent capacity to identify subgroups of individuals who follow distinct patterns (trajectories) during the time of observation, examine the patterns of variation over time, and the possibility to relate different characteristics to each trajectory.

The aims of this study were to describe and compare patterns (trajectories) of LMM (i.e. work disability and unemployment) among young adults with and without CMDs, to elucidate the sociodemographic and medical characteristics that were associated with these various trajectory groups, and highlight potential differences between work disability and unemployment.

## Methods

### Study population

The study base consisted of all individuals between 19 and 30 years who had a main diagnosis of a CMD from inpatient or specialised outpatient mental health care, or had been prescribed antidepressants during year 2007. The first event of either inpatient or specialised outpatient mental healthcare due to a CMD or due to prescribed antidepressant medication during 2007 was considered as the inclusion “event” (cohort entry date). In order to construct a cohort with young adults without previous mental disorders, and hence already at high risk of LMM, we excluded individuals with a record of inpatient or specialised outpatient health care due to mental disorders between 2001 and 2006 and individuals with prescription of antidepressant medication from July 2005 to 31st of December in 2006. In total, 28,989 persons fulfilled the inclusion criteria, and a randomly drawn 25% sample was used in the analyses (*n* = 7245). For comparative reasons, we had in this study a unique possibility to match a comparison group with data on characteristics from a considerable number of sociodemographic and socioeconomic factors. This enabled us to create a comparison group that was very alike the CMD-group, except for the mental disorder. One individual from the general population, without any record of inpatient or specialised outpatient healthcare due to any mental disorder between 2001 and 2007 or antidepressant treatment between July 2005 and 2007, was randomly matched,by the method simple random sampling (SRS) without replacement [20], to each person in the CMD-group. Matching factors included sex, age, educational level, family composition, type of living area and region of birth (*n* = 7245). As this study covers a cohort of young adults, approximately 38% of them were attending the Swedish educational system at baseline, either in upper secondary school or at university. Individuals were, however, entitled to receive benefits due to both sickness absence and disability pension while they were attending the educational system.

### Registers

Register data were available, both retrospectively and prospectively up to 31st December 2013, from the following agencies: 1) *Statistics Sweden*: age, sex, type of living area, educational level, family composition, country of birth, unemployment (annual number of days), sickness absence (annual number of days), disability pension (annual number of months) and emigration from 1990 and onwards; 2) *The National Board of Health and Welfare*: date and cause of inpatient (1973–2013) and specialised outpatient (2001–13) health care; date of death (1961–2013), and prescription of dispensed antidepressant medication (July 2005–13).

### Outcome measures

LMM was defined as: 1) Annual net months with work disability, defined as the sum of net months with sickness absence (calculated from annual days) and net months of disability pension 2004–13 (i.e. three years before, during and six years after the year of the cohort entry date), 2) Annual months (calculated from annual days) enrolled as full-time unemployed, at the Swedish Public Employment Service 2004–13 and 3) Combined LMM, measured as the sum of annual months with work disability and annual months with unemployment. Part-time sickness absence was converted to full-time, i.e. two days on half-time sickness absence equaled one day of full-time sickness absence. One day of sickness absence or unemployment were equal to 1/30 month of benefit.

### Covariates and diagnostics

We used baseline data of records of inpatient and specialized outpatient mental healthcare, i.e. most severe cases of mental disorders. All diagnoses were coded according to the corresponding codes of the International Classification of Diseases, version 10 (ICD-10) [21]. CMDs were defined as a main diagnosis from inpatient or specialised outpatient mental health care in 2007 due to depressive (ICD-10: F32–33), anxiety (ICD-10: F40–42) or stress-related mental disorders (ICD-10: F43) and prescription of antidepressants, based on the Anatomic Therapeutic Chemical (ATC) classification code N06A [22]. Among the CMD-group, the chronologically latest (closest to the incident event) main diagnosis of mental disorders other than CMDs from in- or specialised outpatient healthcare 2004–07 was considered and categorised as: 1) No mental comorbidity/comorbid CMD only, 2) behavioural, emotional and developmental disorders (ICD-10: F50–59, F60–69, F80–89 and F90–99), 3) substance abuse disorders (ICD-10: F10–19), 4) other (than CMDs) affective/anxiety disorders (ICD-10: F30–31, F34–39 and F44–48) and 5) other mental disorders (ICD-10: F00–09, F20–29 and F70–79). Comorbid somatic disorders treated in specialised health care were measured 2004–07 according to all remaining diagnostic ICD-10 codes (ICD-10: A01-E90 and G01-Z99).

Covariates regarding sociodemographic factors were measured at the 31st of December in 2006 and were categorised as: sex, age (19–24 years, 25–30 years), educational level (low (0–9 years of education), medium (> 9–12 years in education) and high (> 12 years in education)), family composition (married/cohabiting and living together without children, married/cohabiting and living together with children, single without children living at home (including children up to 20 years living with parents) and single with children living at home. We can in the database not identify persons over 20 years that still are living with their parents. Further covariates included: type of living area (big cities (Stockholm, Gothenburg and Malmö), medium-sized cities (cities with > 90,000 inhabitants within 30 km distance from the centre of the city), small cities/villages (all remaining cities/villages) and region of birth (Sweden, Western countries, consisting of Europe, North America, Oceania and Non-Western countries consisting of Africa, Asia and South-America). In analyses with regard to work disability, also previous long-term unemployment (no days, 1–179 days and ≥ 180 days annually) was treated as a covariate. In analyses with regard to unemployment, information on previous long-term sickness absence (no days, 1–89 days and ≥ 90 days annually) was included [23, 24]. Length of unemployment and sickness absence was measured during 2006.

### Swedish social insurance regulations

In Sweden, all individuals from 16 years and onwards, with an income above a certain level, can receive sickness benefit. The employer is responsible for payment of the sickness benefit during the first 14 days and this period is not covered in registers from the Social Insurance Agency [25]. Moreover, there is one qualifying day (more days among self-employed) without benefits. Individuals 19–29 years can, due to sickness, receive time-restricted disability pension if the work capacity is reduced or if compulsory education is not completed at 19 years of age. Persons 30–64 years of age can be granted permanent disability pension if they have a permanently impaired work capacity. All individuals over 16 years can be enrolled at the Swedish Public Employment Service. Persons from age 20 can receive basic levels of unemployment benefit without previous income from work. Moreover, unemployed individuals have in Sweden the right to be on sickness absence and receive disability pension if the work ability is decreased due to sickness and have in some instances also the possibility to study when they are unemployed.

### Statistics

In order to identify trajectory groups of LMM, group-based trajectory (GBT) models were used [26]. These methods can elegantly respond to and capture the inherent heterogeneity regarding patterns of LMM among young adults with CMDs. The model can identify subgroups of individuals who follow distinct trajectories during the time of observation, i.e. both before and after baseline. The plotted curves represent the most likely trajectory of work disability and unemployment, and were measured through a procedure developed for SAS by Nagin et al. [26]. Trajectory groups were measured in terms of trends and levels of LMM, i.e. constant, increasing, decreasing and fluctuating trends at low, medium or high levels of work disability, unemployment or the combined measure of LMM. The year of incident CMDs, year 2007, was defined as time point zero (t0) and the patterns of mean number of net months with work disability or mean number of months with unemployment were measured annually from 2004 (t-3) up to 2013 (t6). An individual that died or emigrated during follow-up was included until the year before the event of death or emigration occurred. A zero inflated Poison regression model (zip) was used and a stepwise process of introducing a higher level of complexity (increasing number of trajectories or increase of polynomial order (0–3)) was performed in order to find the best trajectory model. The best model fit according to the Bayesian information criterion (BIC) indicated 9 groups for both work disability and unemployment. There was, however, an overlap of the patterns in different groups and the size of some groups was just a few percent of the population (i.e. limiting the statistical power for the subsequent logistic regression). For these reasons, a model with five groups was chosen according to a previously applied procedure [27]. Moreover, separately analysed GBT models with five trajectory groups were also chosen for the comparison group and for individuals in the CMD-group with regard to LMM as a combined measure of mean number of months of work disability and unemployment.

In addition, multinomial logistic regression was applied in order to elucidate the associations of different sociodemographic and medical characteristics with the identified trajectory groups. All covariates in the multinomial regression analysis were mutually adjusted for each other. A Log-likelihood test was used to describe differences between trajectory groups regarding all covariates. We also evaluated the strength of these associations, i.e. how much the applied variables together were able to explain of the total variance, by using Nagelkerke R^2^. Moreover, we calculated differences in R^2^ for each factor by consecutively excluding one factor from the full model, in order to assess the contribution of each factor in comparison to the full model.

### Sensitivity analyses

Some sensitivity analyses were performed assessing potential differences with regard to: 1) patterns of LMM between a) patients included due to in- or specialized outpatient mental health care and individuals included due to prescription of antidepressants and b) individuals with CMDs included from the three different diagnostic groups, i.e. depressive, anxiety and stress-related disorders and 2) age category.

There were no particular differences in proportions in the trajectory groups between individuals included from in- or specialised outpatient health care and individuals included due to prescribed antidepressant medication neither with regard to trajectory groups of work disability nor with regard to trajectory groups of unemployment. Moreover, we could not find any particular differences between individuals with depressive, anxiety or stress-related disorders with regard to trajectory groups of either work disability or of unemployment.

When the analyses were stratified on age group, the younger age group (19–24 years) followed to a slightly higher extent the constant low trajectory of work disability (64.2%) compared to the older age group (25–30 years, 59.1%). The younger age group followed, however, to a lesser extent the constant low trajectory group of unemployment (32.1%) compared to the older age group (39.9%). When work disability and unemployment were combined, there were no differences between the age groups.

## Results

Most of the individuals in the CMD-group (and due to matching also in the comparison group) were female, between 25 and 30 years, had medium educational level and were living alone and in big cities. The share of immigrants among individuals in the CMD-group was around 14% (Table 1). Compared to the matched comparison group, individuals in the CMD-group had higher levels of all other covariates including previous long-term (> 180 days) unemployment (4.5% vs. 2.9%), previous long-term (> 90 days) sickness absence (9.7% vs. 0.8%) and somatic comorbidity (77% vs. 66%) (data not shown). The Chi^2^-tests revealed that all these differences were significant (*p* < 0.001).

**Table 1:** Characteristics at baseline for the 7245 individuals, 19–30 years, with incident common mental disorders (CMDs), i.e. depressive, anxiety and stress-related disorders, during 2007 (CMD-group)

		CMD-group N (%)
Sociodemographic factors		
Sex	Male	2925 (40.4)
	Female	4320 (59.6)
Age^1^	19–24 years	3385 (46.7)
	25–30 years	3860 (53.3)
Educational level^1^	Low (0–9 years)	1845 (25.5)
	Medium (> 9–12 years)	3523 (48.6)
	High (> 12 years)	1877 (25.9)
Family composition^1^	Married/living together without child at home	202 (2.8)
	Married/living together with child at home	924 (12.8)
	Single without child at home	5729 (79.1)
	Single with child at home	390 (5.4)
Type of living area^1^	Big cities	2861 (39.5)
	Medium cities	2675 (36.9)
	Small cities/villages	1709 (23.6)
Region of birth	Sweden	6240 (86.1)
	Western countries	424 (5.9)
	Non-Western countries	581 (8.0)
Unemployment^2^	No days	5314 (73.3)
	1–179 days	1608 (22.2)
	> 180 days	323 (4.5)
Sickness absence^2^	No days	5435 (75.0)
	1–89 days	1109 (15.3)
	> 90 days	701 (9.7)
Medical factors		
Mental comorbidities other than CMD^3^	No comorbid mental disorder/comorbid CMD only	6284 (86.7)
	Behavioral/emotional/developmental disorders^4^	391 (5.4)
	Substance abuse disorders^5^	337 4.7)
	Other affective/anxiety disorders^6^	139 (1.9)
	Other mental disorders^7^	94 (1.3)
Somatic disorders^8^	No	1654 (22.8)
	Yes	5591 (77.2)

^1^Measured at 31st of December in 2006. Missing education is considered to be low educational level

^2^Measured during 2006

^3^Measured 2004–07 as last main mental diagnosis other than a CMD

^4^International Classification of Diseases version 10 (ICD-10): F50-F59 (behavioural syndromes associated with physiological disturbances and physical factors), F60-F69 (disorders of adult personality and behaviour), F80-F89 (disorders of psychological development), F90-F99 (behavioural and emotional disorders with onset usually occurring in childhood and adolescence)

^5^ICD-10: F10-F19 (mental and behavioural disorders due to psychoactive substance use)

^6^ICD-10: F30-F31 (manic episode and bipolar affective disorder), F34-F39 (persistent, other and unspecific mood disorders), F44-F48 (dissociative, somatoform and other neurotic disorders)

^7^ICD-10: F00-F09 (organic, including symptomatic, mental disorders), F20-F29 (schizophrenia, schizotypal and delusional disorders) and F70-F79 (mental retardation)

^8^All diagnoses except ICD-10 chapter V (mental disorders)

### Trajectory groups of work disability

**Fig. 1:**
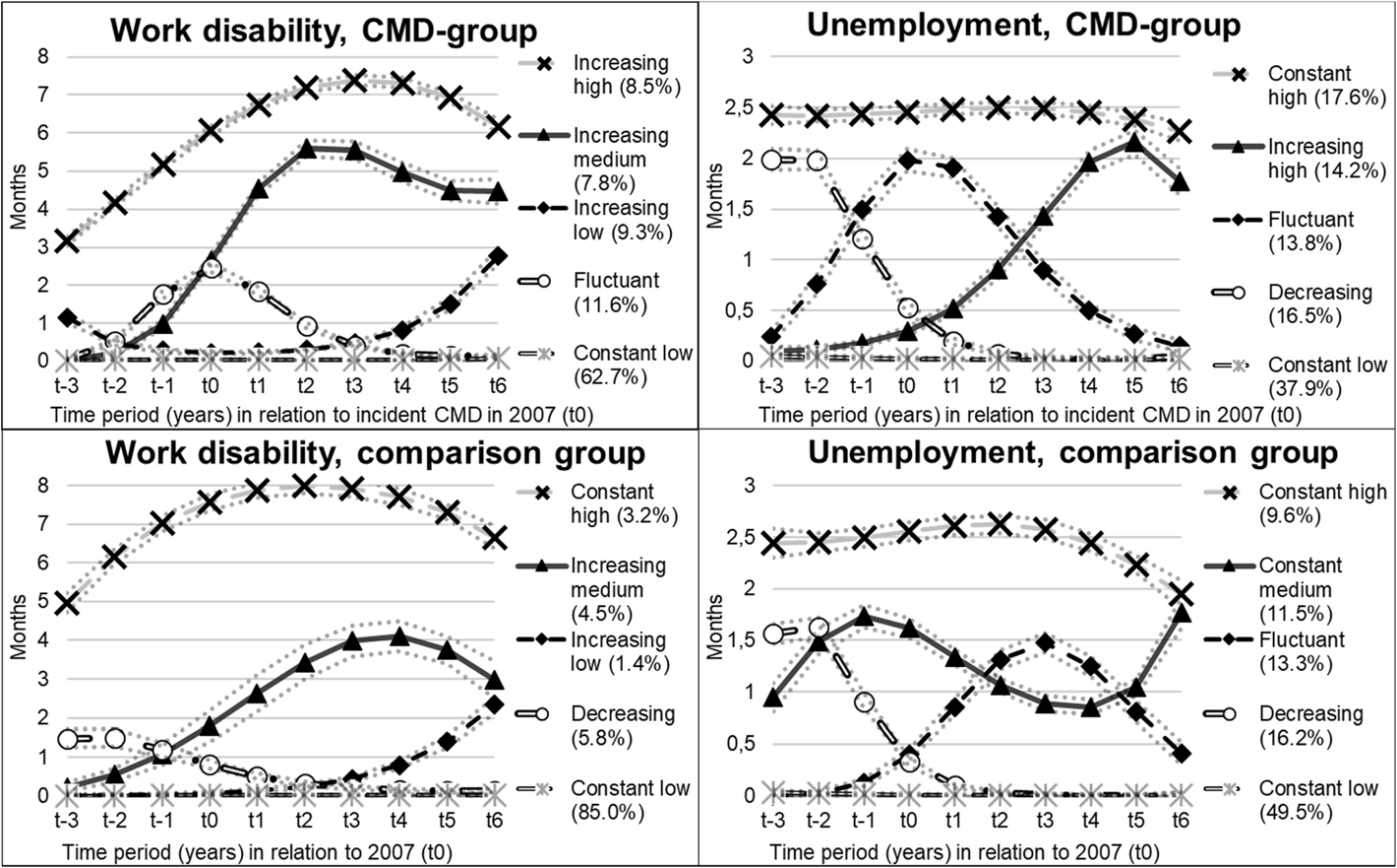
Trajectory groups of work disability and unemployment among the 7245 individuals aged 19–30 years, with an incident common mental disorder (CMD) in 2007 (CMD-group) and the 7245 matched individuals without a mental disorder 2001–07 (comparison group)

Among individuals in the CMD-group, three increasing groups of work disability were identified and were labelled: “increasing high”, with an increasing level of work disability on a high level throughout the follow-up period (8.5%); “increasing medium”, with a rapid increase of work disability around t0 (7.8%) and “increasing low”, with a gradual increase of work disability starting at low levels two years after t0 (9.3%) (Fig. 1). These three groups (together comprising 25.6%) had 6, 4 and 3 months of work disability 6 years after the CMD diagnosis, respectively. The two remaining groups were labelled as “fluctuant”, with a temporary increase of work disability around t0 (11.6%), and “constant low”, with no or very low levels of work disability throughout the whole study period (62.7%). This latter figure compares to 85% of the individuals in the comparison group following the “constant low” trajectory.

In the multinomial logistic regression analyses, all variables were significantly associated with the trajectory groups of work disability (*P* < 0.05, Table 2, Additional file 1: Table S1). The full model explained 17% of the variance between the trajectory groups (Nagelkerke R^2^). The differences in R^2^ indicated that educational level (R^2^ 0.05) and having a mental comorbidity (R^2^ 0.04) were of more importance than other variables in the full model. Individuals with high educational level were overrepresented in the “constant low” trajectory group, whereas there were higher proportions of individuals with a low educational level in the “increasing high”, “increasing medium” and “increasing low” trajectory groups of work disability. The ORs for belonging to the “increasing high” trajectory group of work disability were especially high among individuals with both medium (OR: 4.7) and low (OR: 13.4) educational level compared to belonging to the “constant low” trajectory group of work disability. Moreover, individuals with comorbid behavioural/emotional/developmental disorders (ORs range: 2.8–4.5), other mental disorders (ORs range: 8.9–29.5) and other affective/anxiety disorders (ORs range: 3.0–3.3) had rather high ORs to follow the “increasing high” and the “increasing medium” trajectory groups of work disability in comparison to follow the “constant low” trajectory group of work disability.

**Table 2:** Odds Ratios for belonging to each trajectory group of work disability compared to the reference group (constant low trajectory of work disability) among the 7245 individuals aged 19–30 years, with an incident common mental disorder (CMD) in 2007 (CMD-group)

	Fluctuant vs constant low	Increasing low vs constant low	Increasing medium vs constant low	Increasing high vs constant low	Log-likelihood test (*p*-value)*	R^2^difference**
	OR (CI)	OR (CI)	OR (CI)	OR (CI)		
Sociodemographic factors						
Sex						
Male	1	1	1	1	63.5 (< 0.001)	0.009
Female	1.53 (1.30–1.82)	1.93 (1.60–2.34)	1.18 (0.98–1.44)	1.09 (0.90–1.33)		
Age						
19–24 years	0.39 (0.33–0.47)	0.66 (0.55–0.79)	0.61 (0.50–0.74)	0.48 (0.39–0.58)	155.6 (< 0.001)	0.021
25–30 years	1	1	1	1		
Educational level						
Low (0–9 years)	1.50 (1.17–1.93)	1.91 (1.48–2.46)	2.26 (1.70–3.00)	13.42 (9.47–19.48)	346.5 (< 0.001)	0.052
Medium (> 9–12 years)	2.20 (1.81–2.68)	1.62 (1.30–2.01)	1.95 (1.52–2.52)	4.68 (3.32–6.74)		
High (> 12 years)	1	1	1	1		
Family composition						
Married/living with partner without children at home	1.08 (0.69–1.65)	0.59 (0.33–0.99)	1.15 (0.63–2.01)	1.64 (0.89–2.90)	50.2 (0.012)	0.007
Married/living with partner with children at home	1	1	1	1		
Single/divorced/separated/widowed without children at home	0.68 (0.54–0.84)	0.56 (0.44–0.71)	0.88 (0.66–1.17)	1.16 (0.87–1.57)		
Single/divorced/separated/widowed with children at home	0.72 (0.50–1.01)	0.55 (0.37–0.80)	1.23 (0.82–1.85)	0.75 (0.47–1.20)		
Type of living area						
Big cities	1	1	1	1	32.1 (< 0.001)	0.004
Medium-sized cities	0.77 (0.64–0.92)	0.98 (0.81–1.18)	0.93 (0.75–1.15)	1.29 (1.04–1.60)		
Small cities/villages	0.99 (0.81–1.20)	1.10 (0.88–1.36)	1.18 (0.94–1.49)	1.69 (1.34–2.13)		
Region of birth						
Sweden	1	1	1	1	26.1 (< 0.001)	0.004
Western countries	1.00 (0.73–1.35)	0.71 (0.48–1.02)	0.90 (0.60–1.30)	0.56 (0.36–0.86)		
Non-Western countries	0.69 (0.51–0.92)	0.96 (0.71–1.27)	0.76 (0.54–1.06)	0.51 (0.34–0.72)		
Unemployment						
No days	1	1	1	1	50.9 (< 0.001)	0.007
1–179 days	1.09 (0.91–1.31)	1.29 (1.06–1.56)	1.49 (1.21–1.82)	0.61 (0.48–0.77)		
> 180 days	0.60 (0.37–0.92)	1.20 (0.80–1.74)	0.92 (0.57–1.40)	0.73 (0.48–1.10)		
Medical factors						
Mental comorbidities other than CMD						
No comorbid mental disorder	1	1	1	1	273.2 (< 0.001)	0.037
Behavioural/emotional/developmental disorders	1.61 (1.13–2.27)	1.03 (0.67–1.55)	2.79 (1.99–3.85)	4.48 (3.34–5.98)		
Substance abuse disorders	1.28 (0.88–1.82)	0.86 (0.54–1.30)	1.25 (0.82–1.84)	1.08 (0.72–1.59)		
Other affective/anxiety disorder	1.94 (1.14–3.16)	0.58 (0.22–1.24)	3.01 (1.80–4.88)	3.28 (1.96–5.34)		
Other mental disorders	2.66 (1.01–6.31)	3.15 (1.20–7.43)	8.88 (4.30–18.18)	29.49 (16.46–55.26)		
Somatic disorders						
No	1	1	1	1	58.2 (< 0.001)	0.008
Yes	1.71 (1.40–2.11)	1.66 (1.33–2.09)	1.64 (1.29–2.10)	1.60 (1.27–2.04)		

*Derived from the multinomial logistic regression. All analyses were mutually adjusted for all other variables

**Difference in Nagelkerke R^2^ between full model (R^2^ = 0.17) including tested variable and model without tested variable

In the comparison group, mostly educational level (R^2^ 0.08) was of importance for the differences between the trajectory groups (data not shown).

### Trajectory groups of unemployment

The trajectory groups of unemployment among individuals in the CMD-group (Fig. 1) were named: “constant high”, with high annual levels of unemployment (2–3 months) during the study period (17.6%), “increasing high”, with increasing unemployment during the follow-up period (14.2% and 1–2 months 6 years after diagnosis), “fluctuant”, with a temporary increase in unemployment around t0 (13.8%), “decreasing”, with high levels of unemployment before t0, but decreasing levels during the follow-up period (16.5%) and “constant low”, with low levels of unemployment throughout the whole study period (37.9%). The latter group comprised nearly half (49.5%) of individuals in the comparison group.

In the multinomial logistic regression analyses, all variables, except for medical factors, were significantly associated with the trajectory groups of unemployment (*P* < 0.05, Table 3, Additional file 2: Table S2). Around 12% of the differences between the trajectory groups were explained by the model (Nagelkerke R^2^). Educational level (R^2^ 0.02), type of living area (R^2^ 0.02) and age (R^2^ 0.02) were more important than other variables as indicated by the differences in R^2^ in the full model. Individuals with low educational level had higher ORs of belonging to both the “increasing medium” (OR: 2.3) and “increasing high” (OR: 3.3) trajectory groups of unemployment. Also individuals with medium educational level displayed higher ORs for belonging to the “increasing medium” (OR: 1.9) or “increasing high” (OR: 2.0) trajectory groups of unemployment compared to belonging to the “constant low” trajectory group of unemployment. Moreover, a higher share of individuals who lived outside big cities, had higher ORs for all three “increasing” trajectory groups of unemployment (ORs range: 1.3–2.3) compared to the “constant low” trajectory group of unemployment. The younger age-group (19–24 years) had higher ORs for belonging to the “increasing medium” (OR: 2.2) trajectory group of unemployment compared to belonging to the “constant low” trajectory group of unemployment.

**Table 3:** Odds Ratios (ORs) for belonging in each trajectory group of unemployment compared to the reference group (constant low trajectory group of unemployment) among the 7245 individuals aged 19–30 years, with an incident common mental disorder (CMD) in 2007 (CMD-group)

	Fluctuant vs constant low	Increasing low vs constant low	Increasing medium vs constant low	Increasing high vs constant low	Log-likelihood test (p-value)*	R^2^difference**
	OR (CI)	OR (CI)	OR (CI)	OR (CI)		
Sociodemographic factors						
Sex						
Male	1	1	1	1	56.6 (< 0.001)	0.007
Female	0.94 (0.80–1.10)	0.85 (0.73–0.98)	0.79 (0.67–0.92)	0.58 (0.50–0.67)		
Age						
19–24 years	1.68 (1.43–1.97)	0.94 (0.80–1.09)	2.16 (1.83–2.56)	0.79 (0.68–0.92)	158.3 (< 0.001)	0.021
25–30 years	1	1	1	1		
Educational level						
Low (0–9 years)	2.13 (1.72–2.65)	1.11 (0.89–1.37)	2.28 (1.80–2.90)	3.26 (2.64–4.04)	175.5 (< 0.001)	0.023
Medium (> 9–12 years)	1.35 (1.11–1.63)	1.25 (1.06–1.48)	1.84 (1.50–2.28)	2.00 (1.66–2.43)		
High (> 12 years)	1	1	1	1		
Family composition						
Married/living with partner without children at home	1.44 (0.87–2.32)	1.59 (1.03–2.43)	1.50 (0.87–2.53)	1.30 (0.81–2.07)	43.5 (< 0.001)	0.005
Married/living with partner with children at home	1	1	1	1		
Single/divorced/separated/widowed without children at home	1.27 (1.00–1.63)	1.35 (1.08–1.69)	1.41 (1.09–1.83)	1.38 (1.12–1.72)		
Single/divorced/separated/widowed with children at home	1.91 (1.29–2.80)	1.65 (1.13–2.41)	2.25 (1.51–3.35)	2.70 (1.93–3.80)		
Type of living area						
Big cities	1	1	1	1	134.7 (< 0.001)	0.017
Medium-sized cities	1.24 (1.05–1.47)	1.30 (1.12–1.53)	1.72 (1.44–2.05)	2.01 (1.71–2.36)		
Small cities/villages	1.48 (1.21–1.79)	1.56 (1.30–1.88)	2.33 (1.92–2.84)	2.09 (1.73–2.52)		
Region of birth						
Sweden	1	1	1	1	76.1 (< 0.001)	0.01
Western countries	1.23 (0.89–1.68)	0.85 (0.61–1.17)	1.56 (1.13–2.13)	1.55 (1.16–2.05)		
Non-Western countries	1.81 (1.37–2.39)	1.49 (1.12–1.96)	2.24 (1.69–2.96)	2.57 (2.00–3.30)		
Sickness absence						
No days	1	1	1	1	22.2 (0.005)	0.003
1–89 days	0.75 (0.59–0.93)	1.07 (0.88–1.29)	1.04 (0.84–1.29)	1.12 (0.92–1.36)		
> 90 days	1.15 (0.88–1.50)	1.32 (1.04–1.67)	1.37 (1.05–1.77)	1.43 (1.13–1.81)		
Medical factors						
Mental comorbidities other than CMD						
No comorbid mental disorder	1	1	1	1	18.7 (0.29)	0.002
Behavioural/emotional/developmental disorders	0.91 (0.65–1.24)	1.07 (0.78–1.44)	0.99 (0.72–1.36)	0.86 (0.62–1.18)		
Substance abuse disorders	1.35 (0.94–1.91)	1.33 (0.93–1.89)	1.32 (0.92–1.88)	1.46 (1.06–2.02)		
Other affective/anxiety disorder	0.90 (0.51–1.53)	1.26 (0.77–2.03)	0.81 (0.45–1.40)	1.09 (0.66–1.76)		
Other mental disorders	0.75 (0.39–1.36)	0.93 (0.50–1.64)	0.76 (0.39–1.40)	0.35 (0.16–0.71)		
Somatic disorders						
No	1	1	1	1	5.3 (0.26)	0.001
Yes	1.13 (0.94–1.36)	0.98 (0.83–1.15)	1.19 (0.99–1.44)	1.08 (0.91–1.28)		

*Derived from the multinomial logistic regression. All analyses were mutually adjusted for all other variables

**Difference in Nagelkerke R^2^ between full model (R^2^ = 0.12) including tested variable and model without tested variable

In the comparison group, region of birth (R^2^ 0.03) and educational level (R^2^ 0.06) were of most importance in explaining the association with unemployment, showing that e.g. non-Western immigrants to a higher extent followed the “constant medium” and “constant high” trajectory groups of unemployment (data not shown).

### Trajectory groups of combined LMM

Group trajectory models with the combined measure of LMM, i.e. when summing up number of days with work disability and unemployment, also revealed heterogeneous patterns. In total 11% and 37% of individuals in the CMD-group had 6, and 3 months of LMM 6 years after the diagnosis, while around 53% had no or low levels of LMM at the end of the follow-up period (Additional file 3: Figure S1).

## Discussion

### Main findings

In this large longitudinal study of young adults with CMDs, with an observation period from three years before to six years after an incident diagnosis of a CMD, we revealed considerable heterogeneity in patterns of LMM. Around 26% (*n* = 1859) of the individuals in the CMD-group followed trajectories of “increasing” or “constant high” levels of work disability and 32% (*n* = 2302) followed trajectories of “increasing” and “constant high” unemployment. In the comparison group, just 9% (*n* = 665) followed “increasing” or “constant high” levels of work disability while around 21% (*n* = 1528) followed trajectories of “increasing” or “constant high” levels of unemployment. A lower share of individuals in the CMD-group followed trajectories of “constant low” work disability (*n* = 4546, 63%) or unemployment (*n* = 2745, 38%). This compares to the level of “constant low” work disability (*n* = 6158, 85%) and unemployment (*n* = 3385, 50%) in the comparison group. Moreover, trajectory groups of fluctuant work disability (12%), fluctuant unemployment (14%) and decreasing unemployment (17%) with low levels or no LMM six years after diagnosis were found. While educational level and mental comorbidity other than CMDs discriminated trajectory groups of work disability, educational level, area of living or age determined differences in patterns of unemployment (R^2^_difference_ = 0.02–0.05, *p* < 0.001).

### Trajectory groups of “constant low” work disability and “constant low” unemployment

The majority of individuals in the CMD-group followed the “constant low” trajectory group of work disability and over one third followed the “constant low” trajectory group of unemployment. Compared to the comparison group, there was a much lower share that followed trajectory groups of constant low work disability and unemployment among individuals in the CMD-group, highlighting the difficulties in labor market participation among individuals in the CMD-group. The multinomial logistic regression analyses showed that individuals in the CMD-group with high educational level were to a greater extent found in the “constant low” trajectory groups. Persons with high educational level may have more possibilities to control e.g. their workload and working hours compared to persons with a low educational level [28]. Individuals with low educational level had instead higher probability to be found in trajectory groups of “increasing medium” and “increasing high” work disability as well as in trajectory groups of “increasing high” and “constant high” unemployment. Persons belonging to the “constant low” trajectory groups of either work disability or unemployment may also have had a later onset of the disease, which have allowed them to finish their university education and they might therefore have much better possibilities to get and keep a job. Approximately half of all mental disorders have an onset before mid-teens and around 75% have debuted before mid-twenties [29]. As an adequate education has become of more importance for the chance of getting a job, this might also explain why persons with CMDs more often have problems in staying in employment during adulthood [14, 30, 31].

### Trajectory groups of “increasing medium” and “increasing high” work disability and “increasing high” and “constant high” unemployment

Individuals that are following trajectory groups of “increasing high” work disability (9%) and “constant high” unemployment (18%) were characterised by having high levels of LMM already before the incident diagnoses of CMDs. Almost three times as many individuals in the CMD-group compared with the comparison group followed the “increasing high” trajectory group, which gives an indication of the implications on work participation. The relatively high level of LMM already before the diagnosis might, despite our efforts to create a cohort of individuals without earlier CMD, be an indication of a reversed causal relationship between CMD and LMM, where marginalisation contributes to illness, as found in several other studies [7, 9]. It is, however, likely that some of those individuals had prior treatment for mental disorders in primary health care, where most of the health care visits due to CMDs occur, or did not have any healthcare at all despite having symptoms of CMDs [32]. Worsening symptoms, which in turn led to a visit in specialised health care, may have caused the increasing trend of work disability at the time before baseline. Individuals in the CMD-group that followed trajectory groups of high levels of work disability and unemployment might also have been more affected by aggravating symptoms of CMDs after the diagnosis and that it hence was difficult to stay at work. It may also reflect that individuals in the CMD-group following trajectory groups of increasing LMM have work places with high psychosocial demands, which might worsen the possibilities to remain in employment [33, 34].

Individuals in the CMD-group with comorbidity with other mental disorders, i.e. schizophrenia and psychoses, were to a greater extent found in trajectory groups of “increasing medium” and “increasing high” work disability. The hardship in finding work among persons with severe mental disorders, such as schizophrenia, is well known [35, 36]. Individuals in the CMD-group with comorbid mental disorders had, however, no increased propensity to follow trajectory groups of “increasing” or “constant high” unemployment. One explanation for these findings might be the competing risk of disability pension, i.e. individuals with severe mental disorders are often granted disability pension early in life and are therefore not any longer at risk of unemployment [3].

### Trajectory groups of “fluctuant” work disability and “fluctuant” unemployment

Fluctuant work disability around the time of an incident CMD diagnosis may seem to be the “ideal” pattern. An initial increase of work disability, which is followed by a decrease when e.g. treatment in health care improved symptoms, sickness absence has given the chance to recover and/or rehabilitation measures at the work place have been successful. However, just around 12% of the individuals in the CMD-group followed the trajectory group of “fluctuant” work disability. Combined with the “constant low” trajectory group, around 75% of the whole study population of individuals in the CMD-group had low or no work disability at the end of the follow-up. This also means that around one fourth of persons with CMDs still had persistent levels of work disability as long as six years after the initial diagnosis. This reflects the difficulties of successful rehabilitation and providing stable gainful employment for individuals with CMDs, as also seen in other studies [4]. When combining days of work disability and unemployment in an additional analysis, it turned out that nearly half of the young individuals with CMDs had some level of LMM 6 years after the diagnosis. From a societal perspective this results in a considerable challenge not only for the individuals themselves, but also for the society due to increased costs for e.g. welfare benefits, health care and productivity loss.

### Differences between trajectory groups of work disability and trajectory groups of unemployment

This study adds to the literature by highlighting the heterogeneity of patterns of LMM among young adults with CMDs, i.e. both in terms of patterns of work disability and patterns of unemployment. The most striking difference was that individuals with CMDs to a higher extent followed trajectory groups of high and increasing unemployment than high and increasing work disability. The regulations in the social welfare system in Sweden may be an explanation for these differences. Eligibility for sickness absence benefits presupposes earlier work, but unemployment benefit can be provided on a basic level to persons without earlier income from work if they are enrolled at Swedish Public Employment Service as a job seeker. Young adults without previous work experience may not be eligible for sickness benefit due to social insurance regulations, and disability pension is rather uncommon among young adults [3, 7, 9].

### Strengths and limitations

Strengths of this study were the use of high quality data from Swedish nationwide registers, which allowed large study populations with practically no loss to follow-up. There was no attrition and the registers have good validity, which has been evaluated in several studies [37–40]. Moreover, this study had a long observation period, which allowed us to observe trajectories of both work disability and unemployment during 10 years, both before and after a diagnosis of a CMD. This study had also a unique possibility to match a comparison group with data on characteristics from a considerable number of sociodemographic and socioeconomic factors. This enabled us to create a comparison group that was very alike the CMD-group, and gave us the possibility to put the results into a societal context.

The study had also some limitations worth mentioning. CMDs were defined by inpatient or specialised outpatient mental health care, which mostly reflects medically more serious cases of CMDs. Individuals treated in primary health care were included only if they were prescribed antidepressant medication. We found, however, no major differences between individuals included from inpatient or specialised outpatient health care or individuals who were included due to prescribed antidepressant medication, with regard to trajectories of neither work disability nor unemployment. Moreover, it should be kept in mind that there might be individuals in the CMD-group that were marginalised, but did not receive any social security benefits. Around 22% of the individuals in the CMD-group were economically inactive during the baseline year, meaning that they had no income, neither from work nor from social benefits. This type of marginalisation was not captured by this study. Moreover, the data from the Social Insurance Agency applied here, covers information on sick-leave benefits. This implies that information on sickness absence during the first 14 days in a sick-leave spell was not available. Only 12–17% of the variance of the trajectory groups were explained by our model. Young adults with CMDs are a heterogeneous group and unmeasured factors like life-style, health behaviour and socio economic conditions might be of importance for belonging to a particular trajectory group. Moreover, work environment, type of work etc. has been shown to be connected to sick leave [41]. Also medical factors that we could not measure, as disease severity and treatment strategies, might be of importance for belonging to a special trajectory [42]. Moreover, there are some methodological issues worth to be mentioned. Limiting the number of trajectory groups might decrease the heterogeneity, we chose to do so in order to avoid small group sizes. Group-based trajectory models provides an approximation of the heterogeneity, but this approximation has in many studies given a good estimation of changes in groups over time [26]. There may be differences in labour market participation between persons 19–24 years and persons 25–30 years. Still, the sensitivity analysis revealed that LMM seemed to be rather equal between younger individuals (19–24 years) and older individuals (25–30 years).

## Conclusions

There is considerable heterogeneity with regard to patterns of LMM among young adults with CMDs. Nearly 50% of young adults with CMDs followed trajectory groups of increasing or high persistent levels of either work disability or unemployment throughout the follow-up period. This means that many young adults with CMDs exhibits considerable long-term problems with LMM. Educational level, mental comorbidity and area of living are important factors to take in consideration in order to prevent high and persistent levels of LMM. Further studies elucidating the heterogeneity among individuals with CMDs and investigating additional factors that can explain different patterns of LMM are warranted.

## Additional files

Additional file 1: **Table S1.** Sociodemographic and medical characteristics of trajectory groups of work disability among the 7245 individuals in aged 19–30 years, with an incident common mental disorder (CMD) in 2007 (CMD-group). Description: Distribution of individuals of trajectory groups of work disability. (DOCX 16 kb)

Additional file 2: **Table S2.** Sociodemographic and medical characteristics of trajectory groups of unemployment among the 7245 individuals in aged 19–30 years, with an incident common mental disorder (CMD) in 2007 (CMD-group). Description: Distribution of individuals of trajectory groups of unemployment. (DOCX 16 kb)

Additional file 3: **Figure S1.** Trajectory groups of labour market marginalisation (LMM), i.e. combined work disability and unemployment among the 7245 individuals aged 19–30 years, with an incident common mental disorder (CMD) in 2007 (CMD-group). Description: Trajectory groups of combined labour market marginalisation (LMM), i.e. unemployment and work disability. (DOCX 53 kb)

## Abbreviations

ATC: Anatomic Therapeutic Chemical classification system of drugs
BIC: Bayesian Information Criterion
CMD: Common Mental Disorder, i.e. a depressive, anxiety or stress-related disorders
GBT-model: Group-Based Trajectory model for measuring patterns of LMM
ICD-10: International Classification of Diseases, version 10
LMM: Labour Market Marginalisation, i.e. unemployment and work disability
OR: Odds Ratio
SRS: Simple Random Sampling
