# Supplementary material for: Trajectories of work disability and unemployment among young adults with common mental disorders
Source: BMC Public Health. 2018 Nov 6;18:1228. doi: 10.1186/s12889-018-6141-y (PMC6219052; doi:10.1186/s12889-018-6141-y)
Supplement: Supplementary file 1 — Table S1. Sociodemographic and medical characteristics of trajectory groups of work disability among the 7245 individuals in aged 19–30 years, with an incident common mental disorder (CMD) in 2007 (CMD-group). Description: Distribution of individuals of trajectory groups of work disability. (DOCX 16 kb) [file 12889_2018_6141_MOESM1_ESM.docx]

**Supplementary table 1.** Sociodemographic and medical characteristics of trajectory groups of work disability among the 7,245 individuals in aged 19-30 years, with an incident common mental disorder (CMD) in 2007 (CMD-group)

|  | **Constant low** | **Fluctuant** | **Increasing low** | **Increasing medium** | **Increasing high** | **Log-likelihood test (p-value)*** | **R2 difference**** |
| --- | --- | --- | --- | --- | --- | --- | --- |
|  | **N (%)** | **N (%)** | **N (%)** | **N (%)** | **N (%)** |  |  |
| **All** | 4,546 (62.7) | 840 (11.6) | 675 (9.3) | 565 (7.8) | 619 (8.5) |  |  |
| **Sociodemographic factors** |  |  |  |  |  |  |  |
| ***Sex*** |  |  |  |  |  |  |  |
| Male | 1,941 (66.4) | 276 (9.4) | 189 (6.5) | 223 (7.6) | 296 (10.1) | 63.5 (<0.001) | 0.009 |
| Female | 2,605 (60.3) | 564 (13.1) | 486 (11.3) | 342 (7.9) | 323 (7.5) |  |  |
| ***Age*** |  |  |  |  |  |  |  |
| 19-24 years | 2,300 (68.0) | 264 (7.8) | 286 (8.5) | 252 (7.4) | 283 (8.4) | 155.6 (<0.001) | 0.021 |
| 25-30 years | 2,246 (58.2) | 576 (14.9) | 389 (10.1) | 313 (8.1) | 336 (8.7) |  |  |
| ***Educational level*** |  |  |  |  |  |  |  |
| Low (0-9 years) | 1,013 (54.9) | 154 (8.4) | 183 (9.9) | 168 (9.1) | 327 (17.7) | 346.5 (<0.001) | 0.052 |
| Medium (>9-12 years) | 2,137 (60.7) | 498 (14.1) | 340 (9.7) | 296 (8.4) | 252 (7.2) |  |  |
| High (>12 years) | 1,396 (74.4) | 188 (10.0) | 152 (8.1) | 101 (5.4) | 40 (2.1) |  |  |
| ***Family composition*** |  |  |  |  |  |  |  |
| Married/living with partner without children at home | 115 (56.9) | 34 (16.8) | 17 (8.4) | 17 (8.4) | 19 (9.4) | 50.2 (0.012) | 0.007 |
| Married/living with partner with children at home | 464 (50.2) | 168 (18.2) | 143 (15.5) | 76 (8.2) | 73 (7.9) |  |  |
| Single/divorced/separated/widowed without children at home | 3,758 (65.6) | 581 (10.1) | 470 (8.2) | 424 (7.4) | 496 (8.7) |  |  |
| Single/divorced/separated/widowed with children at home | 209 (53.6) | 57 (14.6) | 45 (11.5) | 48 (12.3) | 31 (8.0) |  |  |
| ***Type of living area*** |  |  |  |  |  |  |  |
| Big cities | 1,830 (64.0) | 366 (12.8) | 261 (9.1) | 213 (7.4) | 191 (6.7) | 32.1 (<0.001) | 0.004 |
| Medium-sized cities | 1,735 (64.9) | 265 (9.9) | 244 (9.1) | 196 (7.3) | 235 (8.8) |  |  |
| Small cities/villages | 981 (57.4) | 209 (12.2) | 170 (10.0) | 156 (9.1) | 193 (11.3) |  |  |
| ***Region of birth*** |  |  |  |  |  |  |  |
| Sweden | 3,909 (62.6) | 721 (11.6) | 574 (9.2) | 486 (7.8) | 550 (8.8) | 26.1 (<0.001) | 0.004 |
| Western countries | 266 (62.7) | 61 (14.4) | 35 (8.3) | 34 (8.0) | 28 (6.6) |  |  |
| Non-Western countries | 371 (63.9) | 58 (10.0) | 66 (11.4) | 45 (7.8) | 41 (7.1) |  |  |
| ***Unemployment*** |  |  |  |  |  |  |  |
| No days | 3,360 (63.2) | 631 (11.9) | 468 (8.8) | 371 (7.0) | 484 (9.1) | 50.9 (<0.001) | 0.007 |
| 1-179 days | 973 (60.5) | 186 (11.6) | 174 (10.8) | 170 (10.6) | 105 (6.5) |  |  |
| > 180 days | 213 (65.9) | 23 (7.1) | 33 (10.2) | 24 (7.4) | 30 (9.3) |  |  |
| **Medical factors** |  |  |  |  |  |  |  |
| *Mental comorbidities other than CMDs* |  |  |  |  |  |  |  |
| No comorbid mental disorder | 4,084 (65.0) | 727 (11.6) | 610 (9.7) | 443 (7.1) | 420 (6.7) | 273.2 (<0.001) | 0.037 |
| Behavioural/emotional/developmental disorders | 176 (45.0) | 44 (11.3) | 27 (6.9) | 53 (13.6) | 91 (23.3) |  |  |
| Substance abuse disorders | 206 (61.1) | 40 (11.9) | 25 (7.4) | 31 (9.2) | 35 (10.4) |  |  |
| Other affective/anxiety disorder | 63 (45.3) | 22 (15.8) | 6 (4.3) | 23 (16.6) | 25 (18.0) |  |  |
| Other mental disorders | 17 (18.1) | 7 (7.5) | 7 (7.5) | 15 (16.0) | 48 (51.1) |  |  |
| ***Somatic disorders*** |  |  |  |  |  |  |  |
| No | 1,222 (73.9) | 132 (8.0) | 104 (6.3) | 91 (5.5) | 105 (6.4) | 58.2 (<0.001) | 0.008 |
| Yes | 3,324 (59.5) | 708 (12.7) | 571 (10.2) | 474 (8.5) | 514 (9.2) |  |  |

* Derived from the multinomial logistic regression. All analyses were mutually adjusted for all other variables.

** Difference in Nagelkerke R^2^ between full model (R^2^ = 0.17) including tested variable and model without tested variable.
