# Supplementary material for: Trajectories of work disability and unemployment among young adults with common mental disorders
Source: BMC Public Health. 2018 Nov 6;18:1228. doi: 10.1186/s12889-018-6141-y (PMC6219052; doi:10.1186/s12889-018-6141-y)
Supplement: Supplementary file 2 — Table S2. Sociodemographic and medical characteristics of trajectory groups of unemployment among the 7245 individuals in aged 19–30 years, with an incident common mental disorder (CMD) in 2007 (CMD-group). Description: Distribution of individuals of trajectory groups of unemployment. (DOCX 16 kb) [file 12889_2018_6141_MOESM2_ESM.docx]

**Supplementary table 2.** Sociodemographic and medical characteristics of trajectory groups of unemployment among the 7,245 individuals in aged 19-30 years, with an incident common mental disorder (CMD) in 2007 (CMD-group)

|  | **Constant low** | **Increasing high** | **Decreasing** | **Fluctuant** | **Constant high** | **Log-likelihood test (p-value)*** | **R^2^ difference**** |
| --- | --- | --- | --- | --- | --- | --- | --- |
|  | **N (%)** | **N (%)** | **N (%)** | **N (%)** | **N (%)** |  |  |
| **All** | 2,745 (37.9) | 1,030 (14.2) | 1,198 (16.5) | 1,000 (13.8) | 1,272 (17.6) |  |  |
| **Sociodemographic factors** |  |  |  |  |  |  |  |
| ***Sex*** |  |  |  |  |  |  |  |
| Male | 981 (33.5) | 394 (13.5) | 483 (16.5) | 420 (14.4) | 647 (22.1) | 56.6 (<0.001) | 0.007 |
| Female | 1,764 (40.8) | 636 (14.7) | 715 (16.6) | 580 (13.4) | 625 (14.5) |  |  |
| ***Age*** |  |  |  |  |  |  |  |
| 19-24 years | 1,143 (33.8) | 595 (17.6) | 501 (14.8) | 642 (19.0) | 504 (14.9) | 158.3 (<0.001) | 0.021 |
| 25-30 years | 1,602 (41.5) | 435 (11.3) | 697 (18.1) | 358 (9.3) | 768 (19.9) |  |  |
| ***Educational level*** |  |  |  |  |  |  |  |
| Low (0-9 years) | 521 (28.2) | 336 (18.2) | 232 (12.6) | 305 (16.5) | 451 (24.4) | 175.5 (<0.001) | 0.023 |
| Medium (>9-12 years) | 1,270 (36.1) | 473 (13.4) | 621 (17.6) | 540 (15.3) | 619 (17.6) |  |  |
| High (>12 years) | 954 (50.8) | 221 (11.8) | 345 (18.4) | 155 (8.3) | 202 (10.8) |  |  |
| ***Family composition*** |  |  |  |  |  |  |  |
| Married/living with partner without children at home | 80 (39.6) | 28 (13.9) | 40 (19.8) | 23 (11.4) | 31 (15.4) | 43.5 (<0.001) | 0.005 |
| Married/living with partner with children at home | 404 (43.7) | 111 (12.0) | 143 (15.5) | 98 (10.6) | 168 (18.2) |  |  |
| Single/divorced/separated/widowed without children at home | 2,157 (37.6) | 828 (14.5) | 957 (16.7) | 818 (14.3) | 969 (16.9) |  |  |
| Single/divorced/separated/widowed with children at home | 104 (26.7) | 63 (16.2) | 58 (14.9) | 61 (15.6) | 104 (26.7) |  |  |
| ***Type of living area*** |  |  |  |  |  |  |  |
| Big cities | 1,296 (45.3) | 406 (14.2) | 468 (16.4) | 304 (10.6) | 387 (13.5) | 134.7 (<0.001) | 0.017 |
| Medium-sized cities | 933 (34.9) | 370 (13.8) | 436 (16.3) | 388 (14.5) | 548 (20.5) |  |  |
| Small cities/villages | 516 (30.2) | 254 (14.9) | 294 (17.2) | 308 (18.0) | 337 (19.7) |  |  |
| ***Region of birth*** |  |  |  |  |  |  |  |
| Sweden | 2,437 (39.1) | 875 (14.0) | 1,058 (17.0) | 840 (13.5) | 1,030 (16.5) | 76.1 (<0.001) | 0.01 |
| Western countries | 156 (36.8) | 60 (14.2) | 52 (12.3) | 64 (15.1) | 92 (21.7) |  |  |
| Non-Western countries | 152 (26.2) | 95 (16.4) | 88 (15.2) | 96 (16.5) | 150 (25.8) |  |  |
| ***Sickness Absence*** |  |  |  |  |  |  |  |
| No days | 2,081 (38.3) | 820 (15.1) | 873 (16.1) | 754 (13.9) | 907 (16.7) | 22.2 (0.005) | 0.003 |
| 1-89 days | 434 (39.1) | 117 (10.6) | 195 (17.6) | 148 (13.4) | 215 (19.4) |  |  |
| > 90 days | 230 (32.8) | 93 (13.3) | 130 (18.5) | 98 (14.0) | 150 (21.4) |  |  |
| **Medical factors** |  |  |  |  |  |  |  |
| *Mental comorbidities other than CMD* |  |  |  |  |  |  |  |
| No comorbid mental disorder | 2,430 (38.7) | 882 (14.0) | 1,035 (16.5) | 848 (13.5) | 1,089 (17.3) | 18.7 (<0.29) | 0.002 |
| Behavioural/emotional/developmental disorders | 140 (35.8) | 58 (14.8) | 66 (16.9) | 65 (16.6) | 62 (15.9) |  |  |
| Substance abuse disorders | 89 (26.4) | 56 (16.6) | 54 (16.0) | 55 (16.3) | 83 (24.6) |  |  |
| Other affective/anxiety disorder | 46 (33.1) | 19 (13.7) | 27 (19.4) | 18 (13.0) | 29 (20.9) |  |  |
| Other mental disorders | 40 (42.6) | 15 (16.0) | 16 (17.0) | 14 (14.9) | 9 (9.6) |  |  |
| *Somatic disorders* |  |  |  |  |  |  |  |
| No | 663 (40.1) | 216 (13.1) | 295 (17.9) | 201 (12.2) | 279 (16.9) | 5.3 (<0.26) | 0.001 |
| Yes | 2,082 (37.2) | 814 (14.6) | 903 (16.2) | 799 (14.3) | 993 (17.8) |  |  |

* Derived from the multivariable, multinomial logistic regression. All analyses were adjusted for all other variables.

** Difference in Nagelkerke R^2^ between full model (R^2^ = 0.12) including tested variable and model without tested variable.
