# Supplementary figures and images for: Trajectories of work disability and unemployment among young adults with common mental disorders
Source: BMC Public Health. 2018 Nov 6;18:1228. doi: 10.1186/s12889-018-6141-y (PMC6219052; doi:10.1186/s12889-018-6141-y)

**
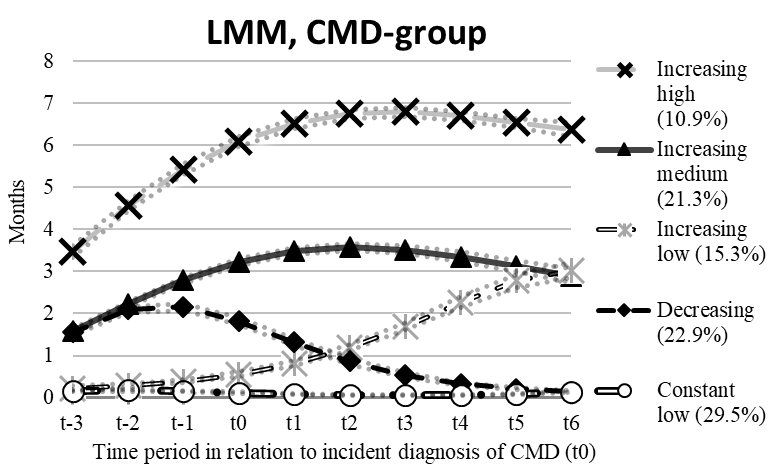
**

Supplement: Supplementary file 3 — Figure S1. Trajectory groups of labour market marginalisation (LMM), i.e. combined work disability and unemployment among the 7245 individuals aged 19–30 years, with an incident common mental disorder (CMD) in 2007 (CMD-group). Description: Trajectory groups of combined labour market marginalisation (LMM), i.e. unemployment and work disability. (DOCX 53 kb) [file 12889_2018_6141_MOESM3_ESM.docx]
